# Endocanalicular transendothelial crossing (ETC): A novel intravasation mode used by HEK-EBNA293-VEGF-D cells during the metastatic process in a xenograft model

**DOI:** 10.1371/journal.pone.0239932

**Published:** 2020-10-21

**Authors:** Federico Armando, Luca Ferrari, Maria Luisa Arcari, Giacomo Azzali, Davide Dallatana, Maura Ferrari, Guerino Lombardi, Matteo Zanfabro, Rosanna Di Lecce, Paolo Lunghi, Ewan R. Cameron, Anna M. Cantoni, Attilio Corradi

**Affiliations:** 1 Department of Veterinary Science, Pathology Unit, University of Parma, Parma, Italy; 2 Department of Medicine and Surgery, University of Parma, Parma, Italy; 3 Istituto Zooprofilattico Sperimentale della Lombardia e dell'Emilia Romagna “B. Ubertini”, Unit of Brescia, Brescia, Italy; 4 Practitioner, 3D Veterinary Printing Project, Parma, Italy; 5 Department of Veterinary Science, Diagnostic Imaging Unit, University of Parma, Parma, Italy; 6 Department of Chemistry, Life Sciences and Environmental Sustainability, University of Parma,Parma, Italy; 7 Centre for Molecular and Translational Oncology, University of Parma, Parma, Italy; 8 School of Veterinary Medicine, University of Glasgow, Glasgow, Scotland; Center for Molecular Biotechnology, ITALY

## Abstract

In cancer metastasis, intravasation of the invasive tumor cell (TCi) represents one of the most relevant events. During the last years, models regarding cancer cell intravasation have been proposed, such as the “endocanalicular transendothelial crossing” (ETC) theory. This theory describes the interplay between two adjacent endothelial cells and the TCi or a leukocyte during intravasation. Two endothelial cells create a channel with their cell membranes, in which the cell fits in without involving endothelial cell intercellular junctions, reaching the lumen through a transendothelial passage. In the present study, ten SCID mice were subcutaneously xenotransplanted with the HEK-EBNA293-VEGF-D cell line and euthanized after 35 days. Post-mortem examinations were performed and proper specimens from tumors were collected. Routine histology and immunohistochemistry for Ki-67, pAKT, pERK, ZEB-1, TWIST-1, F-actin, E-cadherin and LYVE-1 were performed followed by ultrastructural serial sections analysis. A novel experimental approach involving Computed Tomography (CT) combined with 3D digital model reconstruction was employed. The analysis of activated transcription factors supports that tumor cells at the periphery potentially underwent an epithelial-to-mesenchymal transition (EMT)-like process. Topographical analysis of LYVE-1 immunolabeled lymphatics revealed a peritumoral localisation. TEM investigations of the lymphatic vessels combined with 3D digital modelling enhanced the understanding of the endotheliocytes behavior during TCi intravasation, clarifying the ETC theory. Serial ultrastructural analysis performed within tumor periphery revealed numerous cells during the ETC process. Furthermore, this study demonstrates that ETC is an intravasation mode more frequently used by the TCi than by leukocytes during intravasation in the HEK-EBNA293-VEGF-D xenograft model and lays down the potential basis for promising future studies regarding intravasation blocking therapy.

## Introduction

Tumor metastasis is a multi-step process and one of the most relevant events during the neoplastic invasion cascade is the intravasation of the invasive tumor cell (TCi). Several hypotheses about the migratory mechanism of the TCi from the extracellular matrix (ECM) into the lymphatic or blood vessels have been theorized and it is still unclear whether this process involves the TCi in an active or passive manner [1], as to date confirmatory evidence from ultrastructural and three dimensional (3D) approaches has been lacking. In some types of neoplasia, the transendothelial migration of the tumor cell (TC) is assumed to occur via the breakage of the endothelial barrier due to the dissolution of the E-cadherin/β-catenin complex [2] or to apoptosis of the endothelial cells and consequent irreversible retraction of the endothelium [3]. Olah and Glick [4] proposed that during intravasation, diapedesis occurs mainly by a trans-cellular route and lymphocytes pass through a “pore” obtained by a gradual fusion of vesicles secreted by inflammatory cells making it possible to cross the endothelial barrier. According to Uchide and colleagues [5], in Lewis pulmonary tumors, neoplastic cells release the Hete lipid (hydroxyeicosatetraende acid) initiating the retraction of the endothelium, thus facilitating the transendothelial cell migration. According to Miles and colleagues [6], the MCF-7 cell line of the metastatic mammary adenocarcinoma and other tumoral cell lines induce apoptosis of the endothelial cells and consequent irreversible retraction of the endothelium. Studies in zebrafish models [7] and with the MDA- MB-231 mammary tumor cells (M.D. Anderson—metastasis breast cancer) indicate that vascular endothelial growth factor (VEGF) prompts an increase of tumor cell permeation by activation of Ca_2_^+^-dependent signals thought to induce endothelial cell retraction and actin redistribution [8, 9]. Voura and colleagues [10] supported the hypothesis that, during the transendothelial migration, the TCi and the endothelial cell undergo cytoskeletal dynamic changes characterized by alterations of the E-cadherin and platelet endothelial cell adhesion molecule (PECAM). According to Miles and colleagues [6], the Rho-GTPases play an important role in transendothelial migration. Vestweber [11] theorized that, similarly to leukocyte extravasation, the TCi migratory transendothelial process requires the active involvement of the endothelial cell, with the TCi releasing signals responsible for the opening and closure of the interendothelial cell contacts following adhesion to the endothelium. After 40 years of ultrastructural studies on the lymphatic and blood vessel endothelium from different animal species and under different physiological and pathological conditions [12–15], Azzali observed an interesting peculiar behavior of endothelial cells. In particular, an interplay between two adjacent endothelial cells during cell intravasation (TCi or leukocyte) was highlighted. Specifically, two endothelial cells create a channel with their cell membranes and cytoplasm, in which the cell fits in without involving endothelial cell intercellular junctions, reaching the lumen through a transendothelial passage ending the so-called “Endocanalicular Transendothelial Crossing” (ETC) process. Azzali proposed the hypothesis of the ETC subsequently extended to neoplastic cell migration [16]. However, studies exploring the ultrastructural and 3D morphological changes occurring between the neoplastic cell and the endothelial cell during transendothelial migration have been missing. The lack of a dynamic morphological ultrastructural study can be now overcome by combining two approaches: transmission electron microscopy (TEM) microphotographs and a novel approach of 3D virtual model editing. 3D models make possible the explicit visualization of this particular process starting from Born’s technique [17] modified by Werner [18] used to create a 3D wax model, then digitized to produce a reconstructive virtual model. Based on these premises, the aim of the present study is to improve the knowledge on the morphological and ultrastructural changes occurring during neoplastic cell intravasation into lymphatic vessels in a xenograft SCID mouse model, combining immunohistochemistry (IHC), TEM and 3D digital modeling to clarify the endocanalicular transendothelial crossing hypothesized by Azzali. Furthermore, this study aims to quantify this phenomenon in order to establish whether and how frequently ETC is used by the TCi during the intravasation process for the metastatic migration.

## Materials and methods

### Mouse model

Ten 8-week-old female C.B.-17/IcrHantmHsd-Prkdcscid mutant mice (SCID mice), were xenotransplanted with 100 μl of 1x10^7^ HEK-EBNA293-VEGF-D neoplastic cells suspended in Eagle’s Minimum Essential Medium (EMEM), injecting cell suspension in the sub-umbilical region on the right side of the mammary line. Negative controls were included in each mouse by inoculation of 100 μl of EMEM on the fore left side of the mammary line. Mice were housed for 1 week for acclimation, then housed individually in 500 cm^2^ cages in ventilated cabinets with positive pressure and individually identified (S for SCID mice from 1 to 10).

Mice were weighed at 7 days before inoculation, on the inoculation day, and 35 days post-inoculation. The Human Embryonic Kidney-Epstein-Barr Nuclear Antigen293-Vascular Endothelial Growth Factor-D (HEK-EBNA293-VEGF-D) cell line constitutively expressing vascular endothelial growth factor D (VEGF-D) [19] was kindly provided by Prof. Steven Stacker, The Peter MacCallum Cancer Centre, Melbourne, Australia. HEK-EBNA-293-VEGF-D cells were thawed according to standard protocols, seeded at 5x10^4^ cells/cm^2^ and cultured for 1 week in EMEM containing 2 mM glutamine, 1% non-essential amino acids and 10% fetal bovine serum (FBS). On day 7, cells were detached and cell suspension was used for inoculation. The experimental design was approved by the Ethics Committee of the University of Parma, Parma (Italy)—Prot. #54/11 signed on June 14, 2011.

Any other information about the animal model, clinical, and post-mortem examination of the current study are reported as supplementary material.

### Optical and transmission electron microscopy

For optical microscopy, specimens collected from the organs of splanchnic cavities, lymph nodes *inguinales superficiales* and sections of subcutaneous masses were formalin-fixed (10% v/v, pH 7.4), wax-embedded (Bio-Plast 56–58°C, Bio-Optica, Milano, Italy) and 5 μm-thick paraffin sections were set up for histology and IHC. For TEM analysis, a topographic sampling was performed collecting five specimens for each subcutaneous mass section in the following order: specimens 1, 2 and 3 at the periphery, specimen 4 at halfway from periphery to the center and specimen 5 in the center (S1 Fig). A biphasic procedure of fixing/stabilizing intracellular and intercellular viable and non-viable elements was used. Specimens collected were pre-fixed in 1% osmic acid in sodium veronal buffer (pH 7.4) for 10 min., and then cut into pieces using a dissecting microscope (Zeiss binocular optical stereo microscope), ranging 3–4 mm of edge-lengths, and fixed in 1% osmic acid in sodium veronal buffer at pH 7.4 for 2 h.

### Histology

After fixation, 5 μm-thick slides were stained for routine histology using Mayer’s Haematoxilyn-Eosin (H&E).

### Immunohistochemistry

The primary antibodies were titrated according to the manufacturer’s recommendations. Immunohistochemistry was performed as previously described [20] with minor modifications. Briefly, after dewaxing-rehydration, sections were exposed to antigen retrieval, then sections were cooled at room temperature (RT) for 20 min before being soaked into 3% H_2_O_2_ for 12 min. Slides were then rinsed twice in PBS pH 7.4, followed by serum blocking with normal goat serum. First antibody incubation was carried out and slides were then incubated overnight at 4°C. After being washed twice in PBS pH 7.4, slides were incubated for 30’ with a biotinylated goat anti-rabbit or goat anti-mouse IgG antibody. Afterwards, an avidin-biotin-peroxidase (ABC) kit (Vectastain, Elite, ABC-Kit PK-6100, Vector Labs) and 3’3’-diaminobenzidine (DAB) system (DAB-Kit—SK4100, Vector Labs) were used for visualization of antigen-antibody reactions. Nuclei were counterstained with Mayer’s hematoxylin.

For negative controls, the first antibody was replaced by rabbit serum or Balb/c ascitic fluid at corresponding primary antibody protein concentrations. Antibody details are reported in S1 Table. For phospho-AKT(pAKT), phospho-ERK (pERK), zinc finger E-box binding homeobox-1 (ZEB-1), Twist family bHLH transcription factor 1 (TWIST-1), Ki-67, E-cadherin, and F-actin, a semi-quantitative analysis was performed according to the following score: 1 = 0–25%; 2 = 25–50%; 3 = 50–75%; 4 = 75–100% immunopositive cells. The analysis was assessed manually by counting 5 evenly distributed fields at the tumor periphery and 5 fields within the tumor center at 400x magnification using a Nikon Eclipse E800 microscope (Nikon Corporation, Japan) with Nikon PLAN APO lenses and equipped with Camera DIGITAL SIGHT DS-Fi1 (Nikon Corporation, Japan) acquiring pictures with DS camera control unit DS-L2 (Nikon Corporation, Japan) and storing them in an USB device. The normality distribution of the data was evaluated with the Shapiro-Wilk test, followed by the unpaired t test. Statistical significance for each analysis was set at a p-value ≤ 0.05. All statistical analyses were performed with GraphPad Prism version 8.0.1 for Windows (GraphPad Software, La Jolla, CA, USA)

### Transmission Electron Microscopy (TEM)

TEM samples after fixation were treated and stained as previously described by Davalli and colleagues [21] and by Reynolds [22]. Samples were further analyzed using a transmission electron microscope (EM 10A, Carl Zeiss Microscopy GmbH, Jena, Germany) equipped with a 2K‐CCD‐Camera (TRS). For each mouse, 5 peripheral tumor associated lymphatics were chosen and serial ultrathin sections were analyzed in order to quantify the frequency of the canalicular formation in each lymphatic and how many times a TCi or a leukocyte was found within the formed channel. The normality distribution of the data was assessed with the Shapiro-Wilk test and the unpaired t test was further applied. Statistical significance for each analysis was set at a p-value ≤ 0.05. All statistical analyses were performed with GraphPad Prism version 8.0.1 for Windows (GraphPad Software, La Jolla, CA, USA).

### Anatomical wax modeling based on the ultrastructural approach

The technique devised by Born [17] and modified by Werner [18] was used for preparing the 3D wax models. TEM images of a single isolated lymphatic vessel were used for preparing lithographic-like replicas of its section profiles. After deposition of a layer of wax onto the photographic image of the vessel section, each physical contour of the vessel profile could be isolated by surgical excision of the wax and the various contours were piled to reconstruct the 3D wall of the lymphatic vessel under study. This technique can reproduce on a macroscopic scale the canalicular formation used by a neoplastic cell for the transendothelial crossing to the vascular lumen. Azzali further modified Born’s and Werner’s techniques [23] using different colors to improve the distinction between the different endothelial cells involved in the dynamic processes of intravasation [12–15].

The construction of the architecture of the 3D wax model was possible using the serial TEM photographs of tumor-associated absorbing lymphatic vessels; the vessels chosen for modeling had to be in contact with invasive tumor cells (TCi) during the endothelial crossing (transendothelial passage).

According to Azzali [24], the profiles of each endothelial cell component, from TEM photographs, were copied onto tracing paper (60 g) using a pencil (Faber-Castell 2B, Stein, Germany). To facilitate the copy reproduction, an epidiascope with a potent light source was used and TEM images were colored with crayons (Schwan-Stabilo 8740; Schwan-STABILO Schwanhäußer GmbH & Co. KG, Weißenburg, Germany). The sheets were then treated with tetrahydronaphthalene (Merck, 809733), spread out on a lithographic slab, embedded with liquid wax-paraffin, and gauged to a thickness of 1 mm with a steel cylinder at 100°C. After the paper was cooled, the borders of the cells were excised with a lancet, and single serial wax disks were assembled and fixed with paraffin drops to stabilize the 3D model. When properly performed, this wax-disk technique provides the most reliable 3D reconstruction of lymphatic vessels and an accurate stereoscopic view of cellular elements [24]. The 3D wax models were stored in an air-conditioned room at 24°C.

### 3D image reproduction protocol

Computed Tomography (CT) scan of the wax vessel model was performed in order to obtain a Digital Imaging and Communication in Medicine (DICOM) file for a conservative approach of the original model. DICOM files allowed the digital segmentation for generating a digital model of the vessel for digital analysis as plane cuts, highlighting multiple and different “Regions Of Interest” (ROI). Before CT, a radiographic study was performed on the wax vessel model in order to define its radiopacity using computed radiography (Regius 110S, Konica Minolta Healthcare, Tokyo, Japan). CT scan was performed using a single-slice helical CT scanner (Somatom Emotion, Siemens Healthcare, Milan, Italy) with the following protocol: 110 KV, 55 mA, slice thickness: 1.0 mm, pitch: 1.1.

The wax vessel model was placed on the table horizontally, apex to base direction, using radiolucent supports for the best positioning. The whole scan was divided into seven different acquisition windows, in order to acquire the entire wax vessel model volume with the minimum achievable slice thickness. The result was exported in DICOM files which were imported in the 3DSlicer software [25] v.4.5.0 in order to perform the segmentation process and obtain the virtual digital vessel model.

The resulting polygon was exported as a STL file and imported in the Meshmixer software [26] v.11.0.544 in order to analyze the vessel model architecture and structure and to operate combining and repairing where needed.

The final vessel model was a polygon mesh (STL file), resulting from the segmentation of the seven different CT acquisition windows.

The same workflow (CT–DICOM–3DSlicer–Meshmixer) was applied on specifically selected CT images in order to generate new digital vessel models, and allowed the inclusion of color information in the prior grayscale digital reconstruction using the MeshLab software [27] v.2016.12. The color segmentation was performed by following the real wax vessel model color surface.

Specifically, the black/white model is the digital representation of the entire wax model, obtained by seven single CT scan acquisitions. The seven DICOM files were all imported into 3DSlicer to obtain seven “blocks” which were combined as sequence to generate the final model, using an automatic segmentation protocol.

The color model was generated from segmentation of 4 consequent slides from DICOM files (1 mm each slide). The manual segmentation was performed in order to obtain cell profile models, following the real color profile visible on the wax model. For each “step” there are 4 models, highlighted with different color (cyan, green, yellow, red).

## Results

### Immunohistochemical characterization supports an invasive behavior of the HEK-EBNA293-VEGF-D cell line and a mainly peripheral disposition of LYVE-1 immunolabeled lymphatic vessels

**Fig 1:**
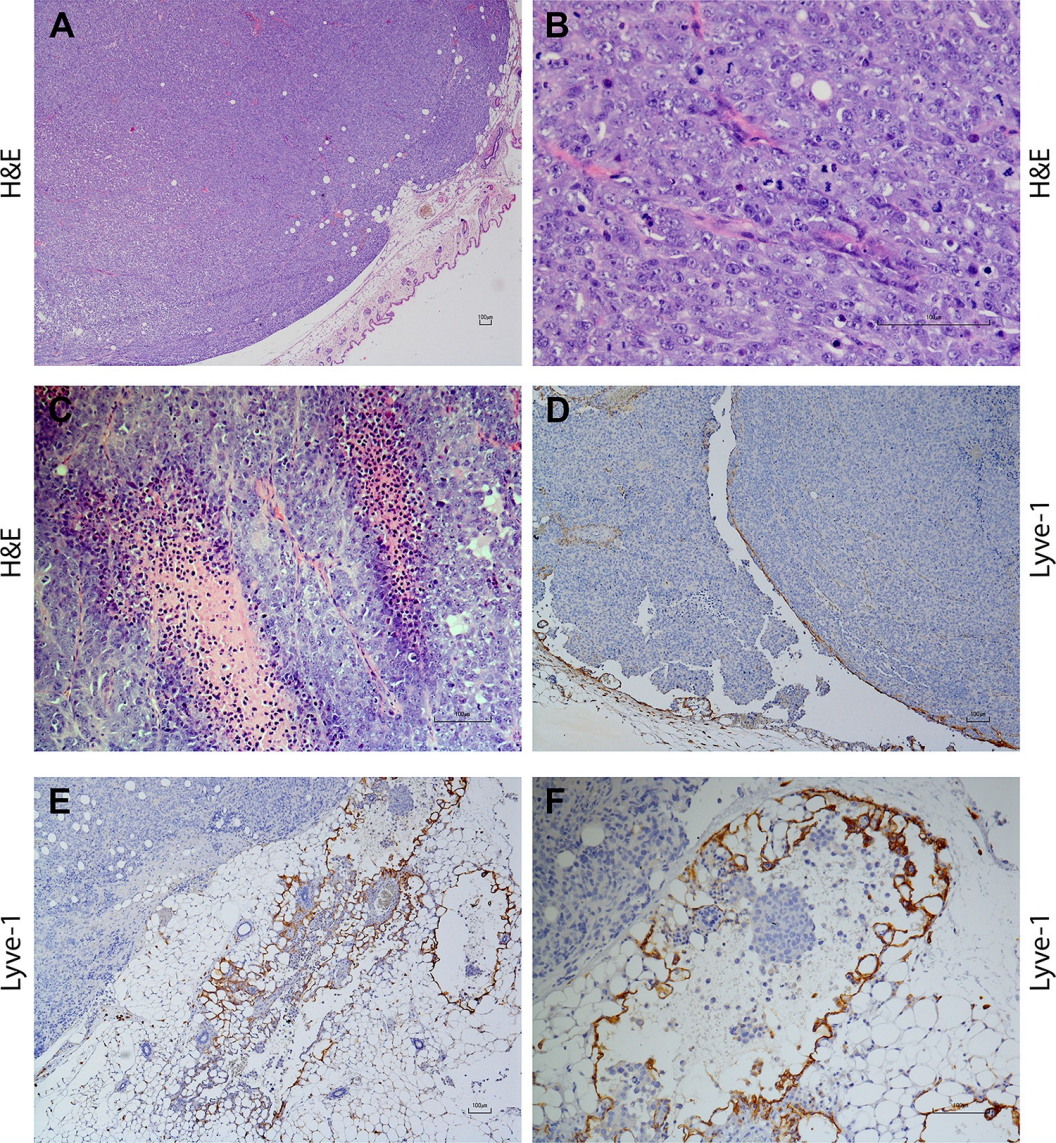
Overview of the histological (A to C) and immunohistochemical (D to F) findings of the HEK-EBNA293-VEGF-D cells xenotransplanted in SCID mice. **A**: Well-defined, partially encapsulated neoplastic sub-cutaneous mass showing locally expansive growth and densely populated by epithelial cells arranged in chords and supported by a moderate fibrovascular stroma (2x, H&E); **B**: polygonal cells with a moderate to high nuclear-cytoplasmic ratio. Round to oval nuclei with vesicular chromatin, and evident nucleoli, round and 1–2 in number, basophilic, and paracentrally located. In this field, high mitotic rate and scattered apoptotic figures are observed among neoplastic cells (40x, Haematoxilyn-Eosin, H&E); **C,** Multifocal intratumoral infiltrates composed by moderate number of neutrophils and fewer lymphocytes embedded in a pale eosinophilic, fibrillar, material (necrosis, 20x, Haematoxilyn-Eosin, H&E). **D, E, F**: Lyve-1 immunolabeled lymphatic endothelial cells (4x, 10x, 20x) localized in peripheral areas of the tumor among neoplastic cells; **F**: Neoplastic emboli were frequently found within Lyve-1 immunolabeled lymphatic vessels (40x).

**Fig 2:**
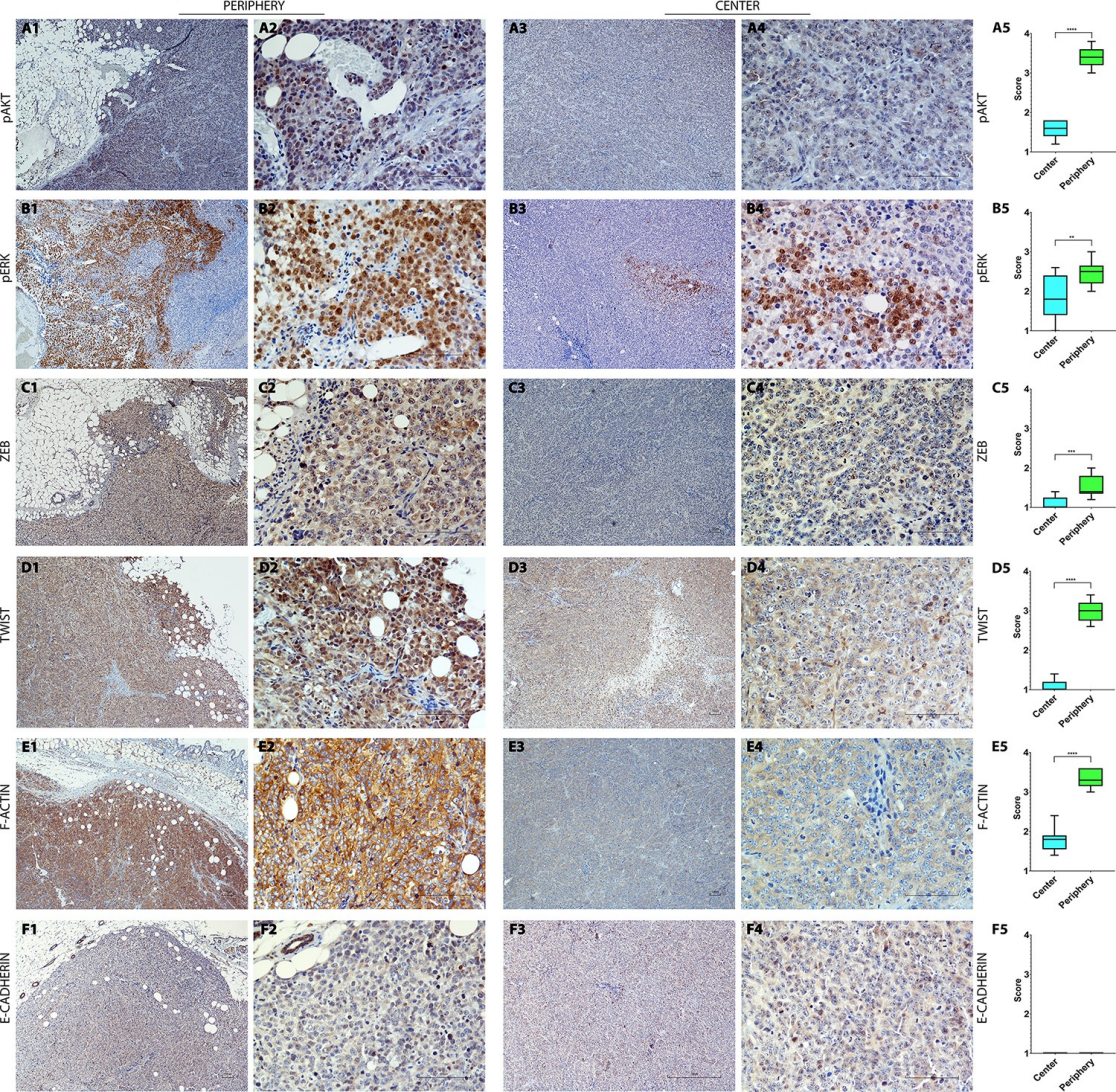
Overview of immunohistochemical characterization (A1-A5 to F1-F5) of the HEK-EBNA293-VEGF-D cells xenotransplanted in SCID mice that supports an invasive phenotype on the periphery of the tumor. **A1-A5:** tumor periphery displaying an increased number (mean score = 3.38) of neoplastic cells with nuclear immunolabeling for pAKT (A1-A2, 4x, 20x) compared to those (mean score = 1.60) at the tumor center (A3-A4, 4x, 20x) as displayed in the box and whisker plots (A5), showing mean values and maximum and minimum values. Significant differences (p ≤ 0.05, t student test) are indicated by asterisks (**** p ≤ 0.0001). **B1-B5:** increased number (mean score = 2.48) of pERK nuclearly immunolabeled neoplastic cells at the tumor periphery (B1-B2,4x, 20x) compared to (mean score = 1.86) tumor central areas (B3-B4, 4x, 20x) as displayed the box and whisker plots (B5), showing with mean values and maximum and minimum values. Significant differences (p ≤ 0.05, t student test) are indicated by asterisks (** p ≤ 0.01). **C1-C5:** tumor periphery displaying an increased number (mean score = 1.52) of neoplastic cells with nuclear immunolabeling for ZEB-1 (C1-C2, 4x, 20x) compared to those (mean score = 1.00) at the tumor center (C3-C4, 4x, 20x) as showed in the box and whisker plots (C5), showing mean values and maximum and minimum values. Significant differences (p ≤ 0.05, t student test) are indicated by asterisks (*** p ≤ 0.001). **D1-D5:** increased number (mean score = 3.00) of TWIST-1 nuclearly immunolabeled neoplastic cells at the tumor periphery (D1-D2,4x, 20x) compared to (mean score = 0.96) tumor central areas (D3-D4, 4x, 20x) as displayed in the box and whisker plots (D5), showing mean values and maximum and minimum values. Significant differences (p ≤ 0.05, t student test) are indicated by asterisks (**** p ≤ 0.0001). **E1-E5:** increased number of membranous to cytoplasmic F-actin immunopositive cells (mean score = 3.32) at the tumor periphery (E1-E2, 4x, 20x) compared to (mean score = 1.78) central tumor areas (E3-E4, 4x, 20x) as displayed in the box and whisker plots (E5), showing mean values and maximum and minimum values. Significant differences (p ≤ 0.05, t student test) are indicated by asterisks (**** p ≤ 0.0001). **F1-F5:** rare cells with a weak cytoplasmic to membranous E-cadherin immunolabeling found both at the tumor center (mean score = 0.22) and periphery (mean score = 0.20) without differences as displayed in the box and whisker plots (F5), showing mean values and maximum and minimum values. Significant differences (p ≤ 0.05, t student test).

The subcutaneous masses were well-demarcated and partially capsulated, with expansive and infiltrative growth, and densely populated by epithelioid cells with morphological changes characteristic of neoplastic cells (Fig 1A). Neoplastic cells were arranged in chords supported by a moderate fibrovascular stroma. Cells were polygonal, sized 28–35 μm, with indistinct cell borders, and the nuclear-cytoplasmic ratio was moderate to high with a moderate to scant, finely granular eosinophilic cytoplasm (Fig 1B). Nuclei were round to oval shaped, sized 21–24 μm, paracentral with vesicular chromatin. From one to two nucleoli were evident, round, sized 2–3 μm, basophilic and paracentrally located. Anisocytosis was moderate, anisokaryosis was high, and high number of cells showed karyomegaly. Mitoses ranged from 5 to 12 per high power field (HPF, 10 fields at 400x). A moderate number of vessels was observed surrounding the tumor periphery and also inside the tumor; these were of variable dimension, in width and caliber. Occasional neoplastic emboli with often more than 10 cells clumped together or less frequently single cancer cells within the lumen of the vessel were observed scattered through the examined sections. In addition, multifocal, scant to moderate infiltrates of inflammatory cells were occasionally detected among neoplastic cells composed by numerous neutrophils, fewer lymphocytes and rare macrophages (Fig 1C). The neoplastic cells partially invaded the underlying skeletal muscle and adipose tissue. Lyve-1 immunolabeling revealed a mainly peripheral distribution of the lymphatic vessels (Fig 1D–1F). Moreover, the proliferation activity given by Ki-67 nuclear immunolabeling (S2 Fig) revealed a high proliferation activity both at the periphery (mean score = 3.54) and within the center of the tumor (mean score = 3.50), without significant difference (p = 0.6470). Furthermore, the transcription factors analysis revealed that pAKT immunolabeled cells at the tumor periphery displayed a significantly increased (p < 0.0001) number of positive cells (mean score = 3.38) expressing activated nuclear pAKT when compared to the tumor center (mean score = 1.60; Fig 2A1–2A5). The number of tumor cells at the periphery expressing nuclearly immunolabeled activated pERK (mean score = 2.48) was significantly increased (p = 0.0038) compared to the central tumor cells (mean score = 1.86; Fig 2B1–2B5). Interestingly, both ZEB-1 and TWIST-1 analysis revealed a significant (p = 0.0009, p < 0.0001, respectively) increased number of immunolabeled neoplastic cell nuclei at the tumor periphery (mean score = 1.52, mean score = 3.00, respectively) when compared to the tumor central areas (mean score = 1.00, mean score = 0.96, respectively; Fig 2C1–2C5 and Fig 2D1–2D5). On the other hand, the cytoplasmic and membranous protein expression analysis showed that both in tumor periphery (mean score = 0.20) and within the center (mean score = 0.22) there was a low number of E-cadherin immunolabeled cells with a weak cytoplasmic to membranous staining, without significant difference (p = 0.8297; Fig 2F1–2F5). Furthermore, semi-quantitative analysis of F-actin immunopositive cells at the tumor periphery showed a significantly increased (p < 0.0001) number of cells (mean score = 3.32) expressing membranous-cytoplasmic F-actin when compared to tumor central areas (mean score = 1.78, Fig 2E1–2E5). Interestingly, numerous F-actin immunolabeled tumor cells were found surrounding or directly within the lumen of peripheral lymphatic vessels (S3 Fig). Taken together, invasion of the underlying soft tissues and the presence of neoplastic emboli may suggest an invasive and early metastatic behavior of the HEK-EBNA293-VEGF-D cell line in the SCID mice xenograft model. Moreover, the high proliferation activity provided by Ki-67 expression together with the increased number of immunolabeled cells for the analyzed transcription factors (pAKT, pERK, ZEB, TWIST) at the tumor invasive front, further support the invasive behavior of the HEK-EBNA293-VEGF-D cell line in the present xenograft model. In addition, invasiveness was also supported by E-cadherin loss and by the increased number of F-actin immunolabeled cells at the tumor invasive front and surrounding the lymphatic vessels, confirming this model as an effective model for studying the tumor early metastatic process, as previously established by Stacker and colleagues [19, 28–31]. IHC findings anatomically localized the tumor-associated lymphatic vessels at the periphery. Further ultrastructural studies focused on the intravasation of the aforementioned invasive cells are necessary to investigate the dynamics of the endocanalicular transendothelial crossing (ETC).

### Interendothelial junctions are not involved during Endocanalicular Transendothelial Crossing (ETC) of the TCi

**Fig 3:**
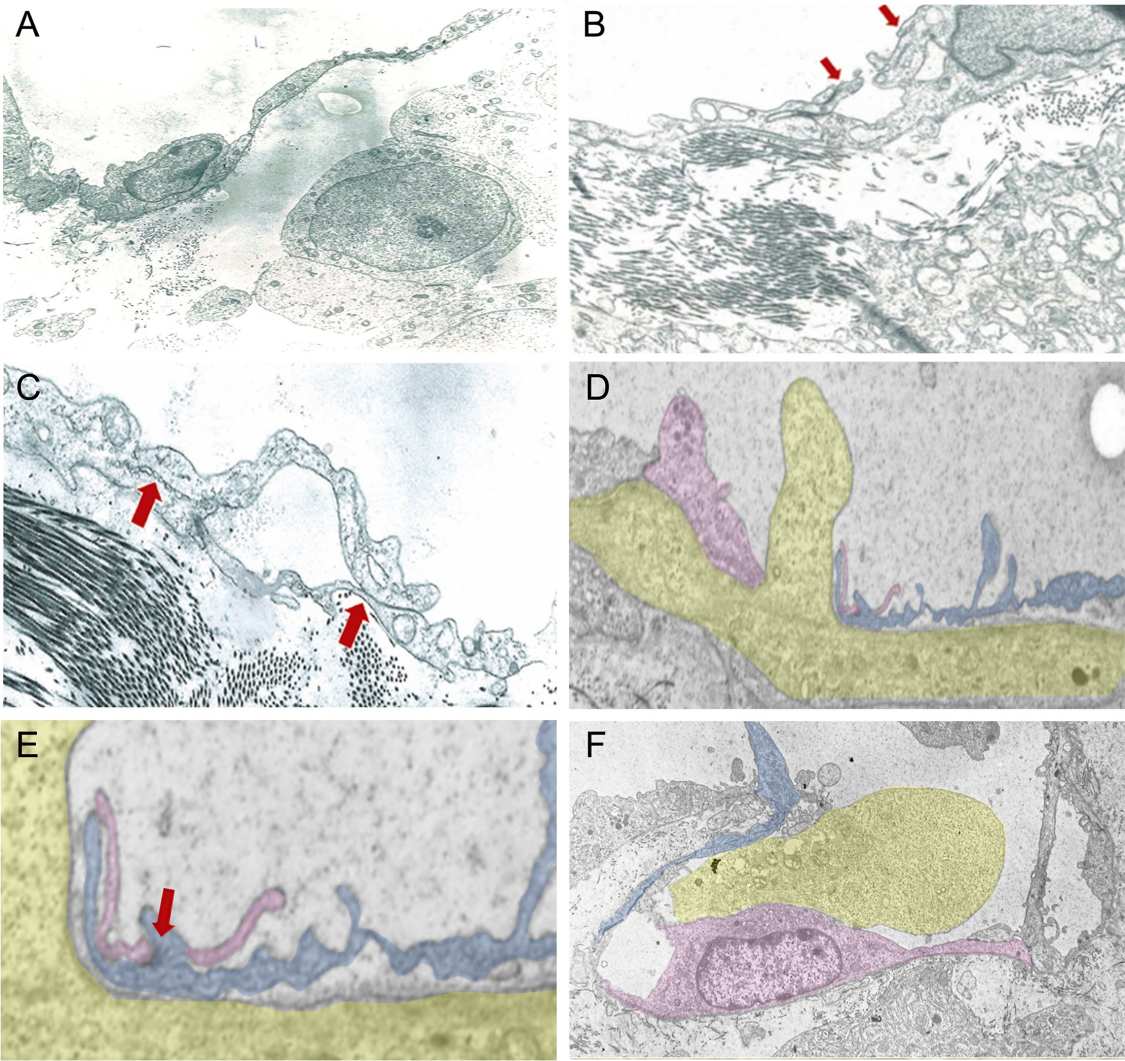
Serial Transmission Electron Microscopy (TEM) micrographs of the main events (A to F) during the endocanalicular transendothelial crossing (ETC) of the invasive tumor cell (TCi). **A**: TEM micrograph of a TCi identified nearby a tumor associated lymphatic vessel (7,500x); **B**: Ultrastructural prodromal sign of the cytoplasmic canalicular formation (finger-like projections) by endotheliocytes (red arrows) (8,500x); **C**: Ultrastructural details of the canalicular formation: the red arrows highlight the presence of tight- and gap-junctions (10,000x); **D**: TEM micrograph of a TCi characterized by a pseudopod-like cytoplasmic protrusion into the neo-formed intraendothelial wall channel (1,200x); **E**: Magnified detail of a TCI intravasation mechanism, not involving interendothelial junction. Red arrow indicates still integer overlapping tight-junctions (1,500x); **F**: TEM micrograph of the last stage of the ETC pathway: a TCi fits into the endothelial endocanalicular wall and enters the vessel lumen (8,500x). Pink and blue: cytoplasms of the two endotheliocytes involved in the channel formation; yellow: cytoplasm of the TCi. The cells in **D**, **E**, and **F** TEM pictures were highlighted in order to better visualize the cell-to-cell overlap or contacts during the TCi intravasation steps. ETC: endocanalicular transendothelial crossing; TEM: transmission electron microscopy; TCi: invasive tumor cell.

The lymphatic endothelium was formed by a monolayer of fairly flattened cells, joined to one another by overlapping and interdigitating intercellular contacts. The endothelial cells were characterized by a cell body that included the nucleus, the common endocytoplasmic organelles, a few lysosomes, free ribosomes and small endoplasmic reticulum tubules. Tumor cells in the extravasal matrix displayed an invasive phenotype (TCi) identified by different morphological changes such as: i) a cellular shape that changed from roundish to elongated; ii) a mostly fragmented nucleus with heterochromatin massed along the peripheral margin of the karyoplasm; iii) a high number of common endocytoplasmic organelles, especially mitochondria; iv) thin ectoplasmic filopod-like and pseudopod-like protrusions (Fig 3A). Those cells displaying the TCi phenotype were frequently found associated with vessel walls. A minimum of 50 to 750 serial ultrathin sections of lymphatic vessels were prepared with one or more TCi in the act of migrating through the endothelial wall in order to examine the process of intravasation. However, only those images that best demonstrated the mechanism of transendothelial crossing were selected. In the early stages of transendothelial migration it was observed that, when the TCi gets close to the endothelial cells, a finger-like cytoplasmic protrusion starting from the endotheliocytes appears (Fig 3B). Often, secondary extensions that originate in the expansions of the cytoplasm of an endothelial cell continue into the interstitial matrix and adhere to the abluminal wall of the adjacent endothelial cell that emanates secondary cytoplasmic extensions fixed by tight and gap junctions (Fig 3C). This behavior, as demonstrated later by 3D reconstructions, precedes the formation of a small channel named intraendothelial channel. After this formation, the TCi, in consequent serial frames, was seen to extend a pseudopod-like cytoplasmic protrusion into the newly formed channel (Fig 3D). The way by which the TCi fits into the endothelial wall shows that a cytoplasmic extension of the TCi itself projects towards the lumen without involving the interendothelial overlapping contacts, which are still fixed by tight- and gap-junctions (Fig 3E). Consequently, the TCi becomes able to slowly reach the lumen, leaving the empty intraendothelial channel (Fig 3F). In the end, the ultrastructural study highlights the main steps of the ETC of a tumor invasive cell underlining how the cancer cell projects its cytoplasm toward the lymphatic vessel lumen, slowly reaching it without breaking down or involving the interendothelial junctions between two adjacent endotheliocytes. To further substantiate our hypothesis, a 3D reconstruction of the aforementioned morphological pictures helps demonstrate the formative process of an intraendothelial channel through which the TCi moves towards the lymphatic vessel lumen.

### 3D image reproduction and virtual animation modeling clarify the ETC theory

Explaining and demonstrating the ETC theory only in 2D might lead to different spatial bias and might result not easy to understand. In order to better explain the morphological changes during the ETC, a representative painted wax 3D model was created allowing further investigation using CT and 3D digital modeling.

TEM analysis of the intravasation process of a TCi allowed us to identify several ETC events in many samples of lymphatic vessels in the different experimental mice. Therefore, the results shown are representative of numerous observed events.

**Fig 4:**
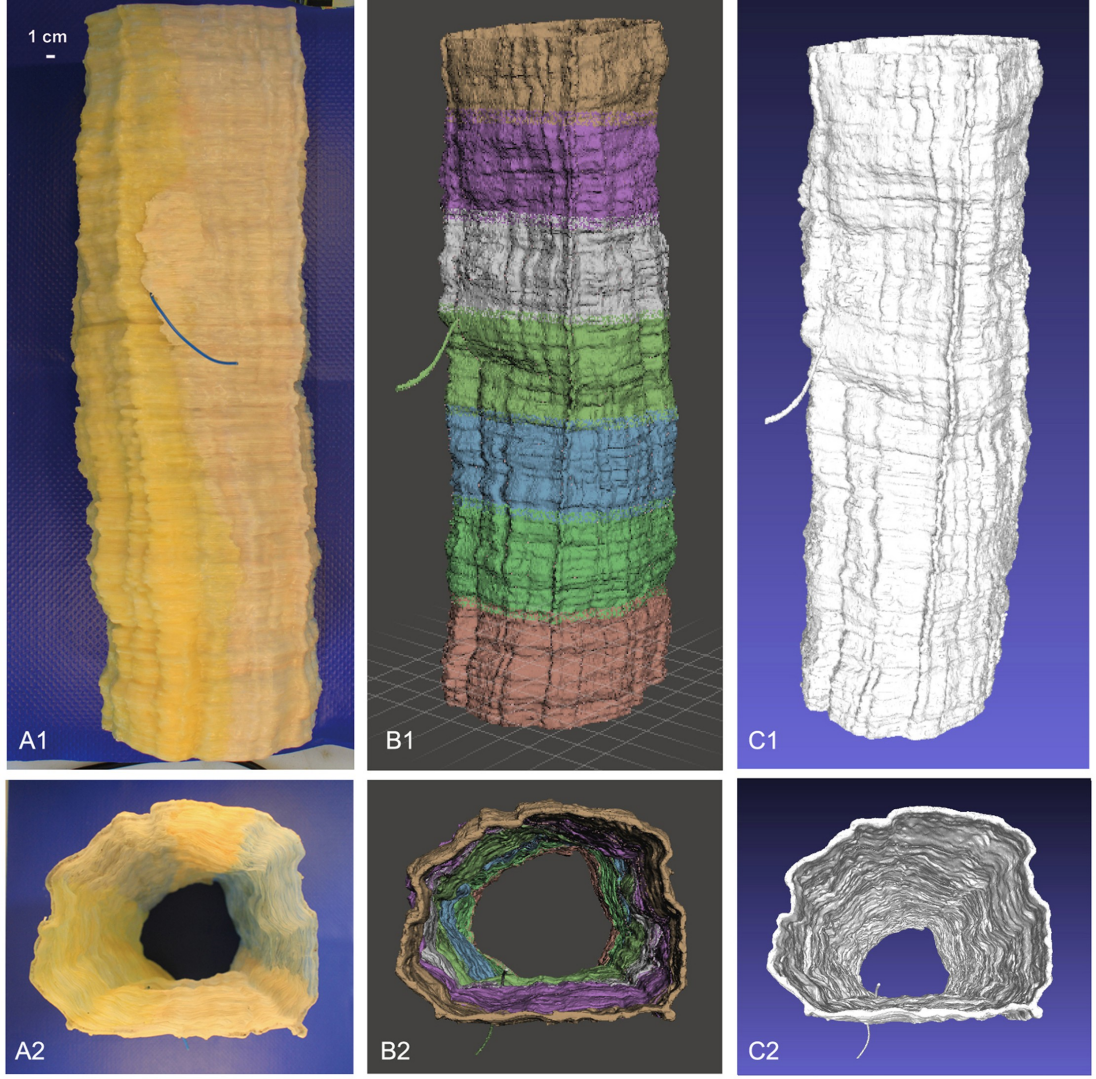
Sequential steps from the TEM-based wax model reconstruction to the DICOM files acquisition and consequential 3D digital model editing (A1-A2, B1-B2, C1-C2). **A1**-**A2**: lateral and top (lumen) view of the wax 3D model of the lymphatic vessel; the blue electric wire crossing the channel shows the intravasation path of a TCi. **B1**-**B2**: lateral and top (lumen) view of the digital reconstruction of the wax 3D model of the lymphatic vessel highlighting the seven CT scan acquisition windows. The result was exported in DICOM files to perform the segmentation process and obtain the virtual digital vessel model. **C1**-**C2**: lateral and top (lumen) view of the digital black/white reconstruction of the TEM-based wax 3D model of the lymphatic vessel. CT: computed tomography; DICOM: Digital Imaging and Communication in Medicine; TEM: transmission electron microscopy.

**Fig 5:**
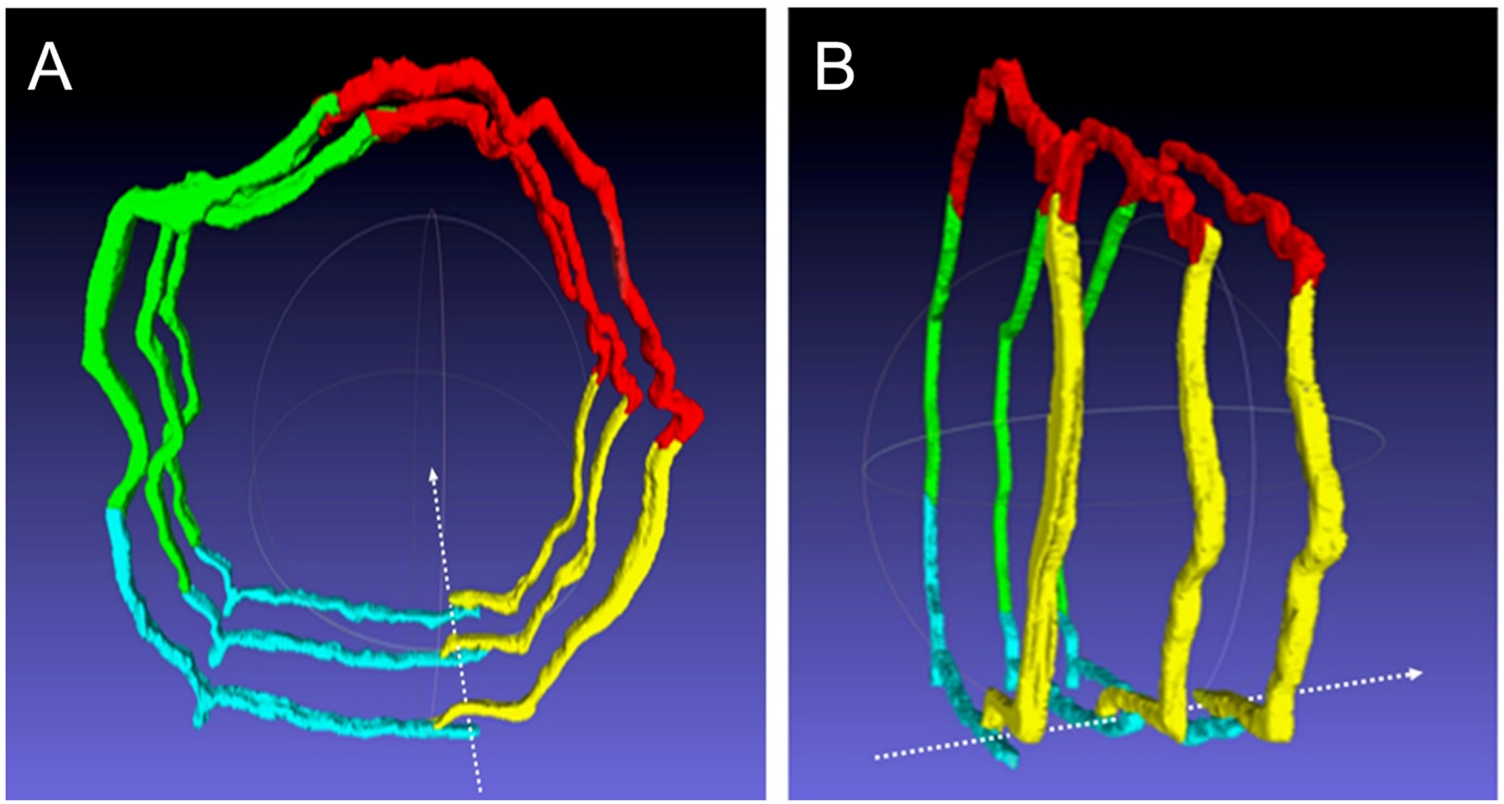
Starting from the black/white model, color models where obtained to better explain the main steps of the ETC phenomenon (A, B). These models together uniquely and properly resembled the endothelial cells behavior during intravasation of a neoplastic cell. Frontal (**A**) and lateral (**B**) views of the color models; the dashed white arrow indicates the path of a TCi crossing the endothelium. The four different endotheliocytes composing the endothelial wall are shown (cyan, green, red, yellow).

The crayon-colored wax 3D model assembling consisted of about 600 wax discs, each one approximately 1 mm thick. The result reproduces a lymphatic vessel segment, in scale 1:10,000, which endothelial wall is crossed by a transendothelial channel (Fig 4A1 and 4A2). The CT scan (Fig 4B1 and 4B2) and the segmentation of the DICOM file from CT scan images generated different models, divided into two main categories: black/white and color models. The black/white model consisted of a total representation of the vessels (Fig 4C1 and 4C2). Starting from the black/white model, cutting and rotating it, color models where obtained to better explain the main steps of the ETC phenomenon (Fig 5A and 5B) These models together uniquely and properly resembled the endothelial cells behavior during intravasation of a neoplastic cell.

**Fig 6:**
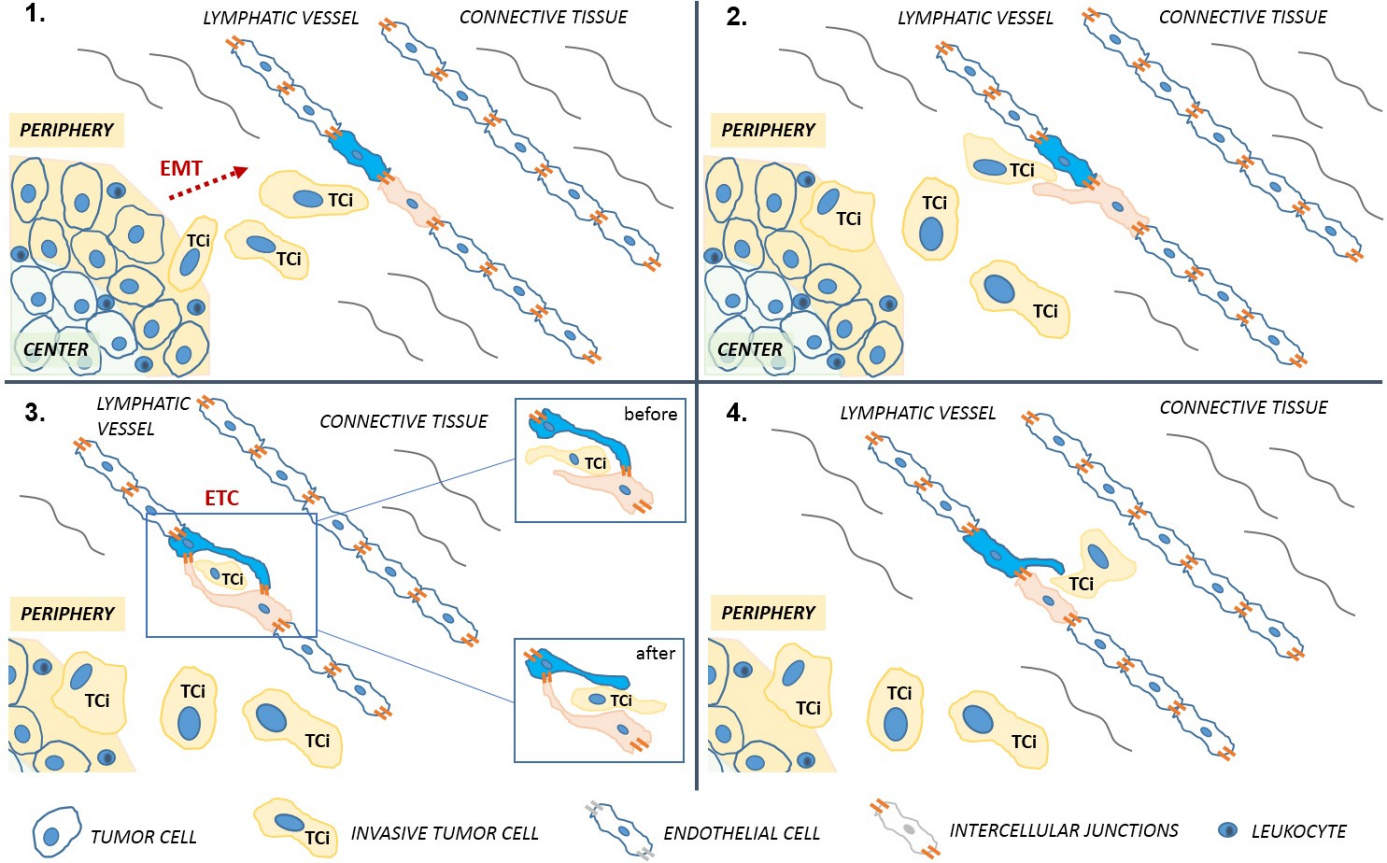
Schematic stepwise representation (A, B, C, D) of the main phases characterizing the endocanalicular transendothelial crossing (ETC). **A:** Invasive tumor cells (TCi) at the tumor periphery undergoing the epithelial-to-mesenchymal transition (EMT) process moving towards the lymphatic vessel. **B:** Early interaction between a TCi and one of the two endothelial cells initiating the ETC phenomenon. The endothelial cell undergoes morphostructural changes typical of such process, namely “finger-like projections”. **C:** Interplay between a TCi and the two endothelial cells during ETC in which the TCi starts entering the early opening due to modifications of the first endothelial cell, which creates a transient inner channel. The TCi begins to exit the channel due to the modifications of the second endothelial cell involved. **D:** A TCi reaching the lymphatic vessel lumen, after complete crossing of the lymphatic endothelial wall without involving endothelial cell intercellular junctions. PERIPHERY: peripheral area of the tumor; CENTER: central area of the tumor.

The dynamic exemplificative interaction between the TCi and the endothelium wall during the ETC is represented in the S2 File. The animation shows the CT scanning of the wax model that perfectly depicts the specific moment and the singular behavior of the endotheliocytes previously described forming the channel. In this animation, the white dot that crosses the channel from an extravasal to luminal direction is the result of the CT acquisition of the electric wire put inside the channel of the wax model, in order to represent the route of the TCi in the act of migrating through it. The 3D virtual model, as Supplementary interactive model (S3 File: https://figshare.com/s/0d01ab9ab08ef56fe9d2) and its animation were able to represent, in an interactive and dynamic mode, the ETC process by a TCi showing this peculiar interaction between TCi and endotheliocytes. The entire process has been further clarified by a stepwise schematic figure (Fig 6).

**Fig 7:**
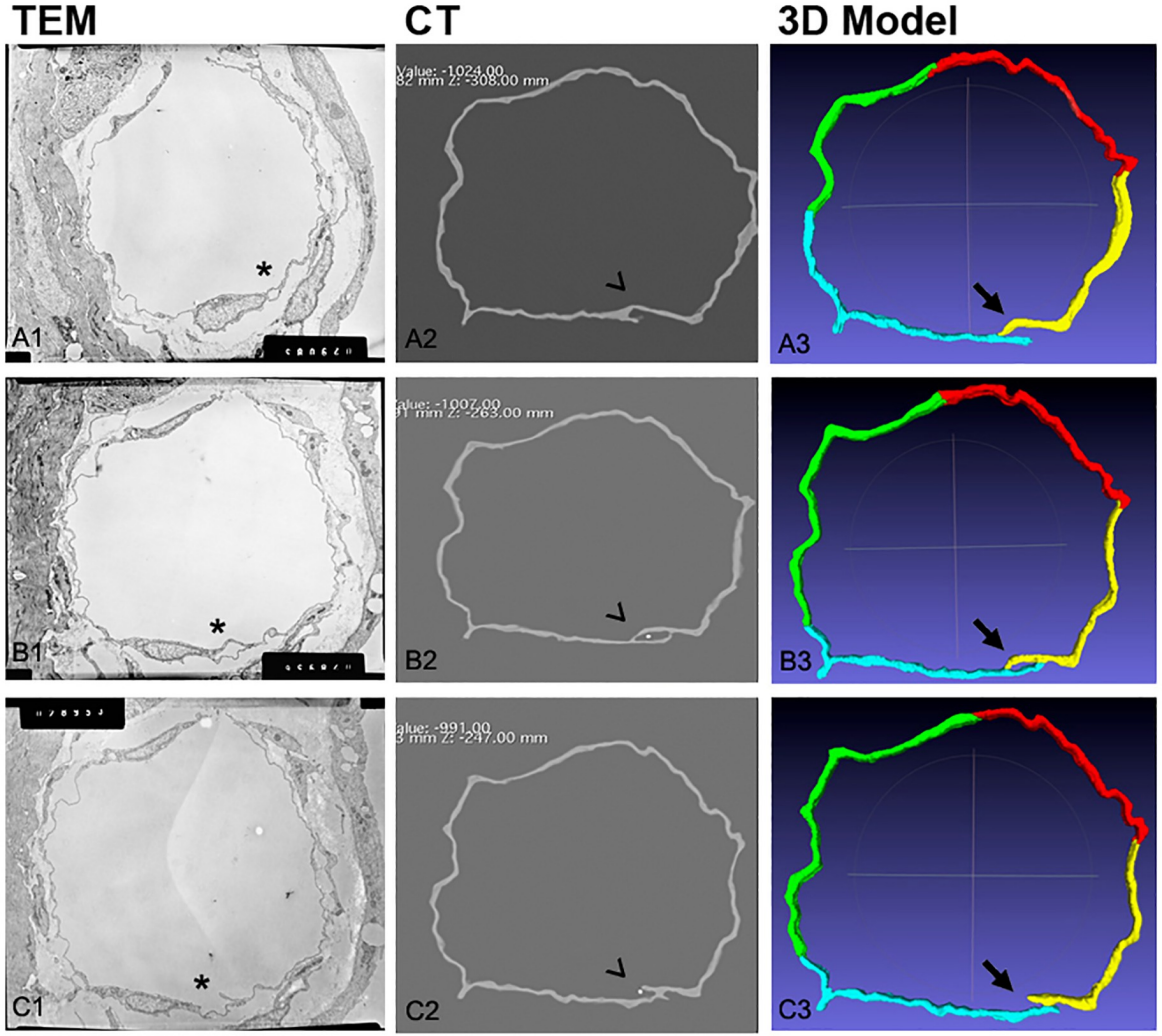
Representative serial sequence combined representation of TEM micrograph–CT scanning–3D digital model at different levels. (A1 to A3; B1 to B3; C1 to C3). In the TEM-CT-3D digital model sequence, from top to bottom and from left to right, three emblematic phases of the intraendothelial channel formation are presented. **A1 to A3**: cytoplasmic evagination (finger-like) opening towards the extravasal space; **B1 to B3**: channel closed with the TCi (white dot) in the lumen; **C1 to C3**: channel opening towards the lumen and TCi in the lumen. TEM: transmission electron microscopy; CT: computed tomography; TCi: invasive tumor cell. The three symbols (*), (^), (↑) indicate the abovementioned main ETC phases shown using the three different techniques.

After confirming the ETC by a TCi using three different techniques as overall summarized in Fig 7, we further quantified the frequency by which the channels in the endothelium are crossed by a TCi or a leukocyte during the metastatic process in our xenograft model.

### Endocanalicular transendothelial crossing is a frequent TCi intravasation mode in tumor-associated lymphatics in the HEK-EBNA293-VEGF-D xenograft model

**Fig 8:**
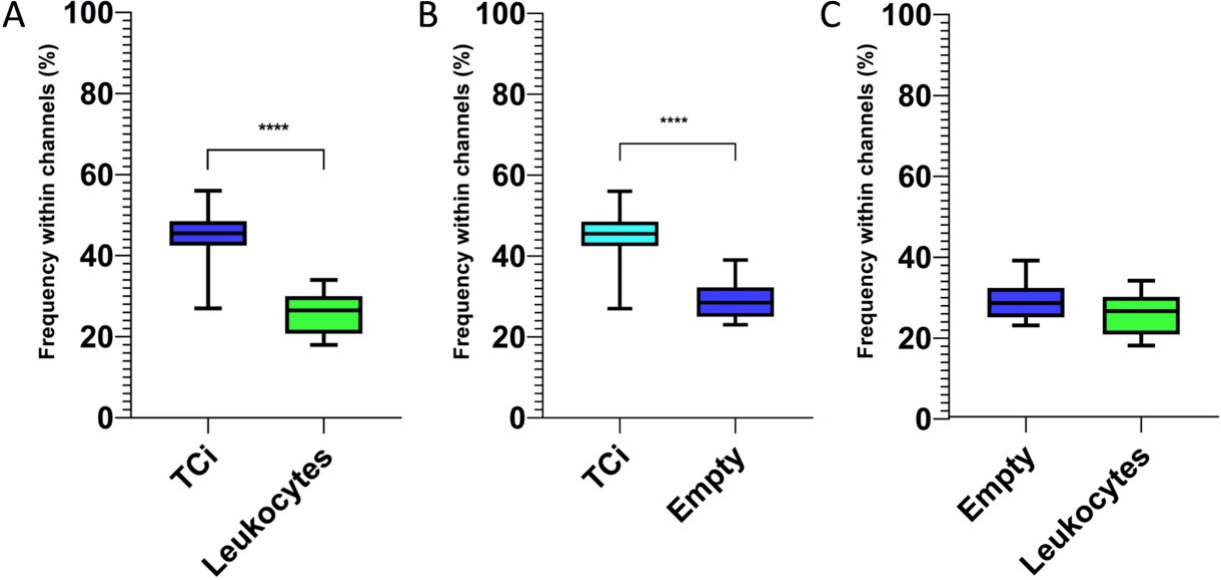
Box and whisker plots with mean values and maximum and minimum values showing how frequently either a TCi or a leukocyte (A to C) were found within the channel during the ETC phenomenon. Optically empty channels were counted as “empty” and compared to the other two categories. **A:** depicts a significant higher number of TCi found within the channel during the ETC when compared to leukocytes; **B:** shows a significant higher number of TCi found within the channel during the ETC when compared to the number of optically empty channels; **C:** reveals no significant differences between the number of leukocytes within the channel during the ETC and the number of optically empty channels. Significant differences (p ≤ 0.05, t student test) are labelled by asterisks (**** p ≤ 0.0001).

For each mouse, 5 peripheral tumor-associated lymphatics were randomly chosen and serial sections were analyzed in order to quantify the frequency of the channel formation and whether ETC was a more frequent phenomenon for inflammatory cells [12–14] or for TCi during the metastatic process [16]. The analysis of tumor-associated lymphatics revealed a mean number of 10.2 channels formed per vessel (range: 2–26). Interestingly, ultrastructural analysis revealed a statistically significant increased number (45%) of channels with a TCi during the ETC (Fig 8A and 8B) process when compared to the number (26%) of channels containing leukocytes (p < 0.0001) or to the number (29%) of optically empty channels (p < 0.0001). No statistically significant difference (Fig 8C) was observed between the number of channels containing leukocytes and the optically empty channels (p > 0.05). In summary, these results of the HEK-EBNA293-VEGF-D xenograft suggest that multiple channels formation can occur within the endotheliocytes of the tumor associated lymphatics along its length. Furthermore, under non-physiological conditions, namely tumor progression, these channels resulted to be more frequently employed by the TCi during the intravasation process than by leukocytes.

## Discussion

The tumor metastatic process is characterized by a complex and wide plethora of events, among which intravasation represents one of the main process. During the invasion phase, invasive tumor cells undergo several transcriptional and morphological changes such as cytoskeletal rearrangements that confer increased motility and changes in shape allowing a successful intravasation [32–35]. In particular, epithelial-to-mesenchymal transition (EMT) is known to be an important step during tumorigenesis and the metastatic process characterized by transcriptional and morphological changes [36, 37]. The results of the present study, namely the increased number of tumor cells at the tumor periphery expressing pAKT and pERK together with low numbers of E-cadherin expressing cells are in line with the results reported by Saba and colleagues in non-small cell lung cancer cells [38]. Interestingly, the aforementioned study suggested that tumors with increased activated pAKT and pERK together with decreased E-cadherin expression are destined to undergo EMT [38]. It has been reported that the EMT process can be initiated by over-expression of transcription factors such as snail family zinc finger 1 (SNAIL), snail family zinc finger 2 (SLUG), TWIST-1, ZEB-1, ZEB-2 [36] and particularly, TWIST-1 is considered as the predominant regulator of EMT [36]. Taken together, we might speculate that HEK-EBNA293-VEGF-D cells underwent EMT-like processes driven by increased number of cells expressing TWIST-1 and ZEB-1 at the tumor periphery, thus acquiring an invasive phenotype. In addition, TWIST-1 has been reported to be up-regulated in a large number of malignant tumor playing a main role in the onset of metastasis by promoting invasiveness [37]. Noteworthy, during an EMT process, ZEB and TWIST expression have been reported to be negatively correlated with E-cadherin expression, and these findings are in accordance with the results of the current study [37]. Among the cytoskeletal rearrangements, polymerization of G-actin into F-actin is one of the most important due to the fact that F-actin is further involved in the formation of the so-called “filopodia” and “invadopodia”, fundamental for the consequent invasion and intravasation process [32–35, 39]. At last, H&E histological findings combined with the assessment of cell proliferation by Ki-67, and the immunohistochemical characterization of nuclear transcription factors, E-cadherin, and F-actin were suggestive of an invasive behavior of the HEK-EBNA293-VEGF-D cells most likely due to EMT-like processes in the TCi at the periphery of the tumor. As a consequence, the HEK-EBNA293-VEGF-D xenograft resulted to be an effective model to study early metastatic processes as previously established by Stacker and colleagues [28–31]. The topographical investigation of LYVE-1 immunopositive lymphatic vessels showed a main peritumoral localization. The absence of tumor associated lymphatic vessels within the tumor center might be explained by an increased mechanical pressure exerted by the neoplastic cells proliferating at the interstitial level that inhibits lymphatics growth [40]. Taken together, these results provided the necessary information to further investigate the ETC theory of a TCi combining preliminary histopathological and immunohistochemical findings with an ultrastructural study. This approach was useful for identifying neoplastic cells at the tumor periphery close to the endothelium or during migration from the matrix to the vessel lumen, thus the sections presenting these features were taken as references to begin the systematic TEM investigations. The TEM study was then performed on serial ultrathin sections to investigate the dynamic interaction between the neoplastic cell and the endothelial cells as described by Azzali [13]. In previous ultrastructural investigations, Azzali showed a different behavior of endotheliocytes under different conditions such as i) physiological (macromolecule passage), ii) inflammation [12–14] and iii) neoplastic metastasis [16]. In these conditions, two adjacent endotheliocytes created a channel with the co-participation of their cell membranes in order to let the neoplastic cells cross the vessel wall and reach the lumen. In the present ultrastructural study, an analogous behavior of endothelial cells was observed. The present work detected the typical different prodromal phases of the cytoplasmic channel formation between endotheliocytes. The starting point is represented by cytoplasmic (finger-like) evaginations emitted by the first endothelial cell, keeping focal contacts (tight-junction) with the adjacent cell that co-participates in the channel formation. Once the tumor cell is engaged in a channel delimited by the cell membrane of the endothelial cells, slowly the second endotheliocyte creates a luminal opening, keeping the focal contacts intact with the adjacent cell and allowing the TCi passage to the lumen of the lymphatic vessel. Ogawa and colleagues [41], studying transcellular channels of endotheliocytes for the vesicle passage, highlighted how it would not be possible to understand the process if the study had been based only on a planar model. Mierke [42] stated that intravasation of a TCi does not need only the impairment of the endothelial layers (breakdown of intercellular contact or induction of apoptosis of the endotheliocytes) but it may be also that the endothelial layers themselves favor intravasation. The last two aforementioned works underline how studying this mechanism in only two dimensions may lead to an incomplete understanding of the process or a different interpretation of the mechanism as discussed by Sage and Carman [43]. In order to better understand and easily explain the ETC theory, confirming the formation of a transendothelial channel, the present investigation employed a novel experimental approach involving a diagnostic technique (CT) and 3D digital model reconstruction. The representative 3D wax model obtained from microphotographs of 700 serial ultrathin sections of the same vessel to reproduce the morphological features of the endotheliocytes forming the channel was converted to a 3D digital model. The 3D digital model allowed us to better understand and explain the morphological modifications of the endotheliocytes. CT scanning provided the same images in serial cross-sections obtaining different frames to produce an animation, offering an easy and dynamic interpretation of the process. The use of an innovative way to reconstruct the intravasation of a TCi clarifies the ETC theory as a novel intravasation mode, visualizing the channel in 3D, rotate and cut the model in a virtual digital space. These combined techniques might be useful for further investigations on the transmigratory behavior of neoplastic cells explaining the stepwise 3D events characterized by morphological modifications and interactions of the invasive tumor cell with the endothelial cell during the intravasation process. The results obtained by using the HEK-EBNA293-VEGF-D cell line model support the reliability of the ETC theory, conceptualized by A.C. and G.A., concerning TCi intravasation during the metastatic process. The present study also aimed to investigate whether the ETC phenomenon observed in this model was an occasional event or a more frequent event during intravasation of a neoplastic cell. The quantitative analysis of the channel formation related to the presence of leukocytes or neoplastic cells within, revealed that, ETC is a common event in the tumor associated lymphatic vessels in this HEK-EBNA293-VEGF-D xenograft model. Furthermore, the detection of either TCi or leukocytes during the ETC phenomenon revealed that this is not only an event that occurs during physiological or inflammatory conditions [12–14] but also under pathological conditions such as tumor growth. However, the mice strain used in this study has various defects related to immune functions such as a decreased number of circulating lymphocytes. Moreover, the HEK-EBNA293-VEGF-D cells may result not to be efficiently immunostimulatory as they are not directly tumor-derived, as supported by the scant and occasional detection of inflammatory infiltrates within the tumor. Considering that VEGF-D not only favors lymphangiogenesis [30] but also the metastatic process [30], and that VEGFs seem to be involved as leukocyte chemoattractants [44–46], VEGF-D constitutively expressed by the HEK-EBNA293-VEGF-D cells might have played a role favoring both the TCi metastatic process and the tumor leukocyte infiltrates in our study. However, it remains unknown whether VEGF-D could have played a role in regulating the specific behavior of the endothelial cells involved in ETC. Despite these aspects, the novel results obtained should be considered encouraging. Future studies will be warranted by using alternative models including more immunostimulatory cell lines and cells of different tumor origin that produce high number of inflammatory infiltrates, allowing to compare those results with the current study. Whether future studies confirm the significantly higher number of TCi detected during the ETC process in comparison with the number of leukocytes, it will be suggestive that this phenomenon is a preferential way for neoplastic cells to metastasize. Confirming the ETC as a mode used by metastatic neoplastic cells for intravasation might lay down the basis to design a complex, targeted and innovative study to provide further scientific findings to understand the pathogenesis of cancer metastasis and to develop new treatment strategies. Future perspectives might also take into account the employment of the digital animation model herein to investigate *in silico* the 3D microenvironment characterized by tumor cells, endotheliocytes and macrophages, as previously described by Zervantonakis and colleagues [47]. Specifically, in breast carcinoma, it would be intriguing to further investigate the existence of a proper tumor microenvironment of metastasis (TMEM) [48] involving the Notch-signaling pathway and Mammalian-enabled (Mena) expression during the intravasation as described by Pignatelli and colleagues [49]. Taking into account the well-documented F-actin structure of the filopodia [34] and the interaction with the ena-vasodilator-stimulated-phosphoprotein (VASP) proteins during filopodia promotion and formation [35, 50] the results by Pignatelli and colleagues might be in accordance with the F-actin over-expression of the metastatic cells of the present study. Future perspectives of the current model may also be focused on isolating tumor-derived endothelial cells, which anti-apoptotic features are sustained by VEGF-D, as described by Bussolati and colleagues [51], and obtaining knock-out cells for intravasation-related genes (e.g. VEGFR-3) to re-inoculate in SCID mice. Using the current TEM ultrastructural approach, this may allow understanding the involvement of such genes in the ETC process. By combining the increasing knowledge on TMEM and signaling pathways with ultrastructural immuno-gold studies focused on the behavior of endotheliocytes that favor the transendothelial passage shown in the present work, it might be possible to propose novel treatment strategies against tumor metastasis via intravasation blocking. In our opinion, the present and future studies should take into account that the proper way to detect ETC seems to be by using serial TEM sections. However, this technique and the molecular characterization of the two endothelial cells directly involved in ETC are mutually exclusive, therefore, this may represent a study limitation. In conclusion, given that the immunohistochemical characterization of the tumor cells at the periphery suggested a potential EMT-like phenomenon and given that the samples obtained from the tumor periphery (on which the serial ultrastructural analysis was performed) showed numerous cells undergoing ETC, we believe that it would be reasonable to hypothesize that the EMT phenomenon is somehow associated with the tumor cell ability to initiate the ETC process. This might be most likely due to cell rearrangements during EMT as suggested by a study mimicking the flow of invasive-metastatic breast cancer cells undergone EMT through constricted microcapillaries [52]. Moreover, the present work suggests that the ETC phenomenon is more frequently used by tumor cells than leukocytes during intravasation in the HEK-EBNA293-VEGF-D xenograft model.

### Post-mortem examination

Mice were euthanized at 35 days post-inoculation and necropsies followed by proper tissue sampling were immediately performed. Euthanasia was performed by a trained person in compliance with the AMVA Guidelines 2007 for mice [53] under the continuous monitoring by the same person performing the procedure, until confirmations of euthanasia was over. Euthanasia was performed with intraperitoneal injection of ketamin followed by vertebral dislocation. Subcutaneous masses were cut in two sections and submitted to IHC and TEM as summarized in S1 Fig. The organs of the splanchnic cavities were examined to identify gross lesions visible and/or recognizable by stereomicroscopy (4x) (Wild M3Z Heereberg, Switzerland). Histology was performed collecting specimens from splanchnic organs and lymph nodes (Lnn), in particular the right and left *Lnn*. *inguinales superficiales* [54].

### Animal experiment, clinical and post-mortem examination

The care and use of animals were conducted in accordance with the principles outlined in the current guidelines published by the National Institutes of Health’s Guide for the Care and Use of Laboratory Animals. SCID mice were found to be an efficient experimental animal model: they were susceptible to tumor growth and developed solid subcutaneous masses. Mice showed a healthy physical status during the experimental period and fodder and drink water consumptions were regular. The recorded weights were within the standard ranges throughout the study, according to the growth curves of each genetic strain provided by the ENVIGO research models [55]. All mice transplanted with HEK-EBNA293-VEGF-D cells developed tumors while no evidence of any lesions was ascertained in the site of the contralateral mammary line inoculated with an EMEM solution. Ten mice were euthanized and necropsies were performed at 35 days post-inoculation. The subcutaneous masses were solid, oval, superficially located, not anatomically fixed and well-defined. The tumor were parallel to the skin, close to or in contact with the dermis, which was always intact and not edematous. The cut surface of the masses appeared homogeneous, from elastic to firm. At euthanasia, the subcutaneous masses ranged from 0.8 to 1.45 cm of diameter. The organs of the splanchnic cavities were examined *in situ* with no gross pathology recognizable to the naked eye and confirmed by stereomicroscopy examination (4x).

## Supporting information

S1 Fig Schematic representation of the tumor mass processing for histology/immunohistochemistry (IHC) and transmission electron microscopy (TEM) analyses. At 35 days post-inoculation of the HEK-EBNA293-VEGF-D cell line, necropsies of the SCID mice were performed and the subcutaneous mass from each mouse was divided into two halves; one section was used for histology and IHC analyses, while five specimens of 0.3 cm (white circles) from the other section were chosen for TEM analysis and sampled in the following order: specimens 1, 2 and 3 at the periphery, specimen 4 at halfway from periphery to the center, and specimen 5 in the center.
(TIF)

S2 Fig Diffuse proliferation activity of HEK-EBNA293-VEGF-D cells xenotransplanted in SCID mice (A to E). **A-D:** Tumor periphery displaying high number (mean score = 3.54) of Ki-67 nuclear immunolabeled neoplastic cells (4x, 20x) that do not differ from tumor center (mean score = 3.50) proliferation activity (4x, 20x); **E:** box and whisker plots showing mean values and maximum and minimum values that do no highlight significant differences between tumor center and periphery. Significant differences (p ≤ 0.05, t student test).
(TIF)

S3 Fig F-actin immunolabeled cells found surrounding peripheral vessels or directly within the vessels (A to B). A-B: Often scattered throughout the tumor periphery, F-actin immunopositive neoplastic cells surrounding hematic and lymphatic vessels (20x, 20x).
(TIF)

S1 Table Details of the antibodies and procedures used for immunohistochemistry. (DOCX)

S1 File (MOV)

S2 File Dynamic representation of the TCi during ETC. The animation shows the CT scanning of the TEM-based wax model that depicts the specific moment and the singular behavior of the endotheliocytes previously described forming the channel. In this animation, the white dot that crosses the channel from an extravasal to luminal direction is the result of the CT acquisition of the electric wire put inside the channel of the wax model created from serial TEM microphotograph, in order to represent the route of the TCi in the act of migrating through it.
(MOV)

S3 File 3D interactive model of a tumor associated lymphatic vessel. The 3D digital model represents an interactive and dynamic mode to represent in 3D the reciprocal positions of the endotheliocyte wall and the TCi during the endocanalicular transendothelial crossing (ETC). https://figshare.com/s/0d01ab9ab08ef56fe9d2.
(U3D)
