# Supplementary figures and images for: Endocanalicular transendothelial crossing (ETC): A novel intravasation mode used by HEK-EBNA293-VEGF-D cells during the metastatic process in a xenograft model
Source: PLoS One. 2020 Oct 21;15(10):e0239932. doi: 10.1371/journal.pone.0239932 (PMC7577447; doi:10.1371/journal.pone.0239932)

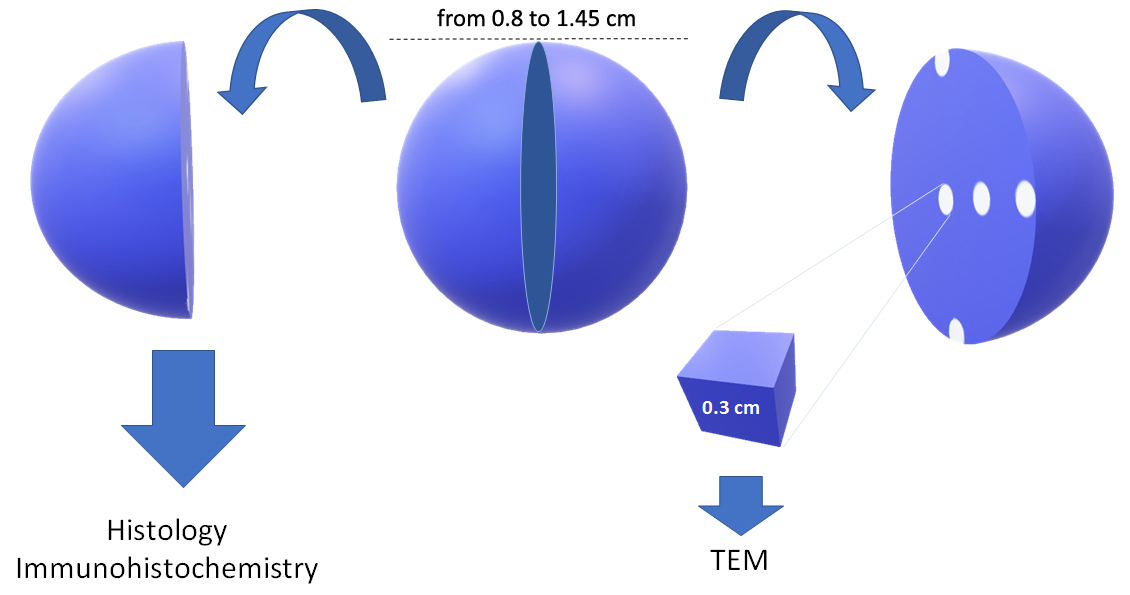

Supplement: S1 Fig — At 35 days post-inoculation of the HEK-EBNA293-VEGF-D cell line, necropsies of the SCID mice were performed and the subcutaneous mass from each mouse was divided into two halves; one section was used for histology and IHC analyses, while five specimens of 0.3 cm (white circles) from the other section were chosen for TEM analysis and sampled in the following order: specimens 1, 2 and 3 at the periphery, specimen 4 at halfway from periphery to the center, and specimen 5 in the center. (TIF) [file pone.0239932.s001.tif]

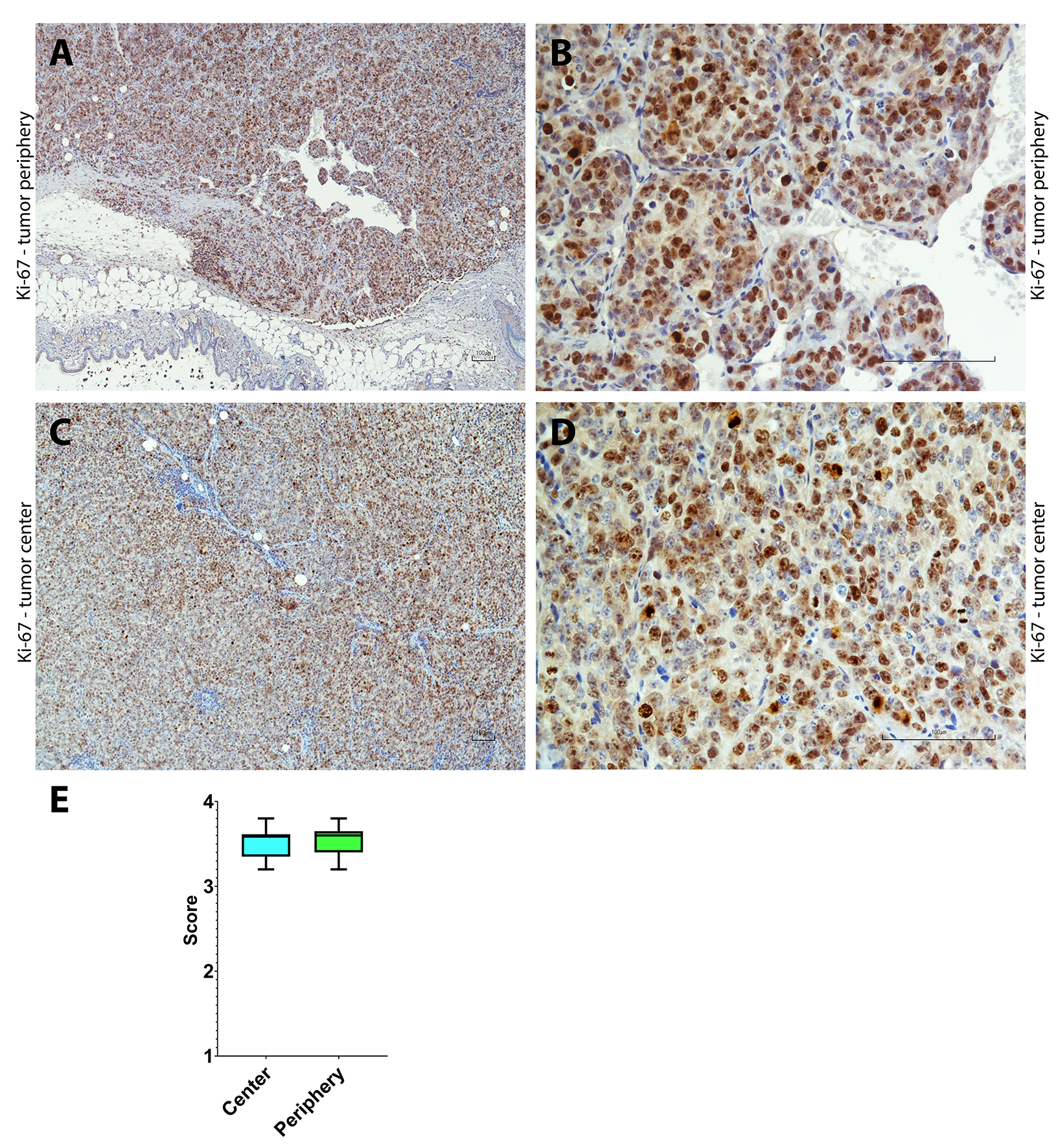

Supplement: S2 Fig — Diffuse proliferation activity of HEK-EBNA293-VEGF-D cells xenotransplanted in SCID mice (A to E). A-D: Tumor periphery displaying high number (mean score = 3.54) of Ki-67 nuclear immunolabeled neoplastic cells (4x, 20x) that do not differ from tumor center (mean score = 3.50) proliferation activity (4x, 20x); E: box and whisker plots showing mean values and maximum and minimum values that do no highlight significant differences between tumor center and periphery. Significant differences (p ≤ 0.05, t student test). (TIF) [file pone.0239932.s002.tif]

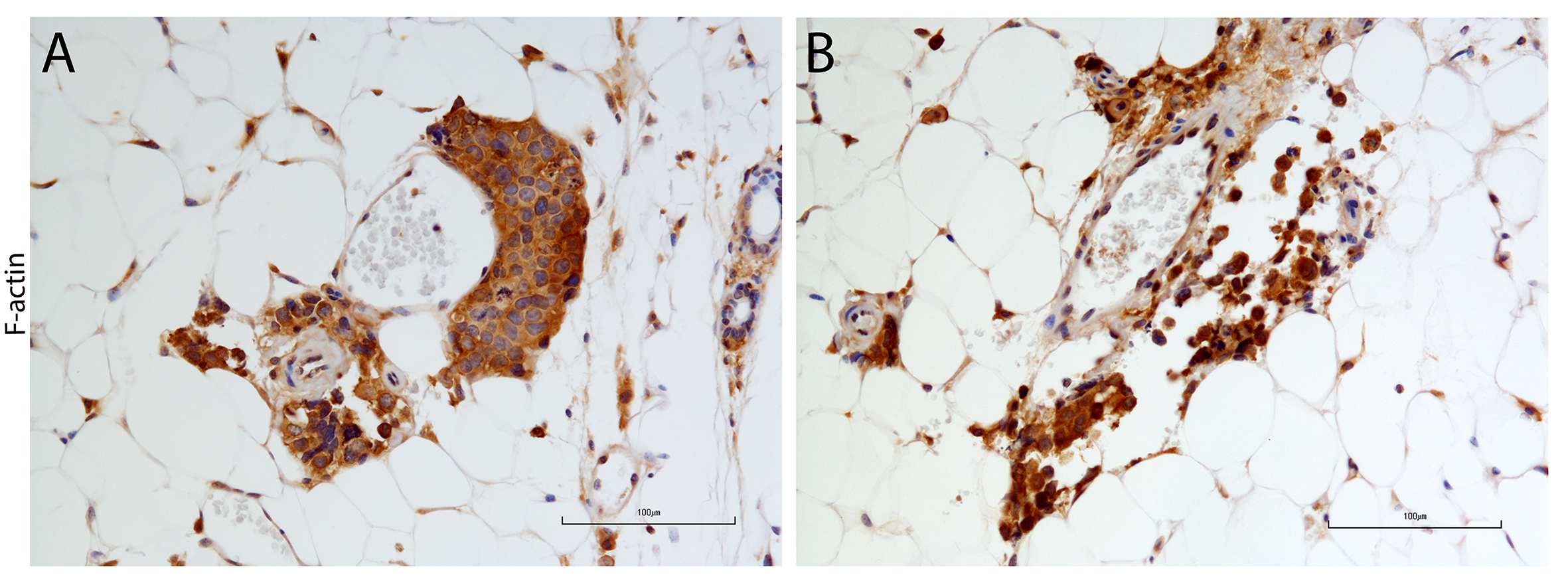

Supplement: S3 Fig — F-actin immunolabeled cells found surrounding peripheral vessels or directly within the vessels (A to B). A-B: Often scattered throughout the tumor periphery, F-actin immunopositive neoplastic cells surrounding hematic and lymphatic vessels (20x, 20x). (TIF) [file pone.0239932.s003.tif]
