# Supplementary material for: Endocanalicular transendothelial crossing (ETC): A novel intravasation mode used by HEK-EBNA293-VEGF-D cells during the metastatic process in a xenograft model
Source: PLoS One. 2020 Oct 21;15(10):e0239932. doi: 10.1371/journal.pone.0239932 (PMC7577447; doi:10.1371/journal.pone.0239932)
Supplement: S1 Table — (DOCX) [file pone.0239932.s004.docx]

**S1 Table .** Details of the antibodies and procedures used for immunohistochemistry.

| Target antigen | Antibody  details/clone | Blocking serum | Heat Induced Epitope Retrieval (HIER) | Primary antibody dilution | Secondary antibody  (1:200) | Positive control |
| --- | --- | --- | --- | --- | --- | --- |
| Ki-67 | Monoclonal mouse anti-human, IgG, clone MIB-1 (Dako) | Goat | Microwave 400W,  3 cycles,  5 min. each, sodium citrate buffer, pH 6.0 | 1:75 | biotinylated goat anti-mouse IgG  (BA-1000 - Vector Labs)  1:200 | Mouse lymph node |
| pAKT (phospho- AKT) | Monoclonal rabbit anti-human, IgG, (D9E) XP™ (Cell Signaling Technology) | Goat | Microwave 400W,  3 cycles,  5 min. each, sodium citrate buffer, pH 6.0 | 1:100 | biotinylated goat anti-rabbit IgG  (BA-1000 - Vector Labs)  1:200 | Prostate adenocarcinoma from TRAMP mice |
| pERK (phospho- ERK) | Monoclonal rabbit anti-human, IgG, (D13.14.4E) XP™  (Cell Signaling Technology) | Goat | Microwave 400W,  3 cycles,  5 min. each, sodium citrate buffer, pH 6.0 | 1:200 | biotinylated goat anti-rabbit IgG  (BA-1000 - Vector Labs)  1:200 | Prostate adenocarcinoma from TRAMP mice |
| ZEB-1 (zinc finger E-box binding homeobox-1) | Polyclonal rabbit anti-human, IgG,  LS-C31478  (LSBio) | Goat | Microwave 400W,  3 cycles,  5 min. each, sodium citrate buffer, pH 6.0 | 1:200 | biotinylated goat anti-rabbit IgG  (BA-1000 - Vector Labs)  1:200 | Mouse Liver |
| TWIST (Twist family bHLH transcription factor-1) | Polyclonal rabbit anti-human, IgG,  Orb329955  (biorbyt) | Goat | Microwave 400W,  3 cycles,  5 min. each, sodium citrate buffer, pH 6.0 | 1:800 | biotinylated goat anti-rabbit IgG  (BA-1000 - Vector Labs)  1:200 | Mouse liver |
| E-cadherin (epithelial- cadherin) | Monoclonal mouse anti-human, IgG2a, clone 36/E-Cadherin  (BD transduction laboratories) | Goat | Microwave 400W,  3 cycles,  5 min. each, sodium citrate buffer, pH 6.0 | 1:100 | biotinylated goat anti-mouse IgG  (BA-1000 - Vector Labs)  1:200 | Mouse skin |
| F-actin  (polymeric filaments) | Polyclonal rabbit anti-mouse, IgG, bs-1571R (Bioss) | Goat | Microwave 400W,  3 cycles,  5 min. each, sodium citrate buffer, pH 6.0 | 1:250 | biotinylated goat anti-rabbit IgG  (BA-1000 - Vector Labs)  1:200 | Mouse skeletal muscle |
| Lyve-1  (lymphatic  vessel endothelial cells) | Polyclonal rabbit anti-mouse, IgG,  103-PA50 (Relia Tech) | Goat | Microwave 400W,  3 cycles,  5 min. each, sodium citrate buffer, pH 6.0 | 1:200 | biotinylated goat anti-rabbit IgG  (BA-1000 - Vector Labs)  1:200 | Mouse small intestine |
